# Frequency-Aware Refinement Network with Multi-Scale Fusion for Remote Sensing Change Detection

**DOI:** 10.3390/s26113538

**Published:** 2026-06-03

**Authors:** Xu Zhang, Yue Du, Zeyu Zhang, Kaihua Zhang

**Affiliations:** 1 School of Digital Low-Altitude, Suzhou Polytechnic University, Suzhou 215104, China; 2 Information Center, Suzhou Polytechnic University, Suzhou 215104, China; 91822@jssvc.edu.cn; 3 Department of Applied Mathematics and Computer Science, Duke Kunshan University, Suzhou 215316, China; zeyu.zhang@dukekunshan.edu.cn; 4 School of Automation, Southeast University, Nanjing 210096, China

**Keywords:** remote sensing, change detection, frequency-aware refinement, coarse-to-fine, multi-scale

## Abstract

Remote sensing change detection (RSCD) identifies land cover variations by comparing bi-temporal images. However, conventional methods relying solely on RGB domain information often fail to distinguish changed objects from visually similar backgrounds, especially in complex scenarios. To overcome this limitation, we propose a frequency-aware refinement network (FARNet) that follows a coarse-to-fine strategy. In the first stage, we design a frequency-aware module (FAM) that learns frequency domain information to identify the blurred boundaries of changed objects that resemble the background, enabling coarse localization of potential change regions. In the second stage, recognizing that high-resolution RGB domain details provide richer spatial information than frequency-domain features, we design a refinement fusion module (RFM) that leverages these RGB details to correct and refine segmentation boundaries, ensuring precise detection. Finally, edge loss is applied to preserve high-frequency details, enhancing the precision of change detection. Extensive experiments on benchmark datasets demonstrate that FARNet significantly outperforms existing methods, achieving superior accuracy and robustness in complex change detection scenarios.

## 1. Introduction

Remote sensing change detection (RSCD) aims to identify changes between two images of the same geographic location captured at different moments. This technology has significant value in urban planning, agricultural monitoring, and ecological assessment [1,2,3]. With the significant progress made in satellite imaging systems, the change detection community has gained substantial data resources and technical support, greatly promoting technological progress. Currently, change detection technology includes both machine and deep learning-based methods.

Traditional machine learning methods were commonly employed for change detection before the widespread adoption of deep learning. These methods can be broadly categorized into pixel-based and object-based approaches. Pixel-based methods rely on direct comparisons of individual pixel values between bi-temporal images to detect changes. They generate difference images and create change maps based on predefined thresholds. Common techniques include simple pixel comparison [4], ratio pixel comparison [5,6], and pixel transformation [7,8,9]. These methods are computationally simple and cost-effective, as they do not require complex feature extraction or model training. However, they are sensitive to environmental factors such as illumination changes and shadows, which can alter spectral information and increase misclassification. Additionally, pixel-based methods often ignore spatial context, potentially causing isolated pixels to be incorrectly identified as changes, thus increasing the false alarm rate. Object-based methods improve change detection by incorporating spatial context. They segment images into meaningful objects and extract features such as edges, textures, and shapes to identify changes. By analyzing objects rather than individual pixels, these methods can reduce false alarms. Object-based methods are often combined with machine learning algorithms, including Support Vector Machine (SVM) [10], Random Forest (RF) [11], and K-Nearest Neighbors (KNNs) [12]. While they generally achieve higher detection accuracy than pixel-based methods by leveraging prior knowledge, such prior knowledge may not always be readily available, and the robustness of these methods can vary across different scenarios.

Deep learning methods have emerged as a dominant approach in change detection, offering superior accuracy and robustness compared to traditional techniques. These methods can handle complex changes in imagery without requiring handcrafted features or special conditions. Convolutional neural networks (CNNs) excel at extracting high-level representations from temporal image pairs. For instance, Daudt et al. [13] proposed U-Net-based networks for change detection. However, deeper networks increase parameter counts and computational costs. Liu et al. [14] addressed this by replacing standard convolutions with depthwise separable convolutions [15], reducing both parameters and computation. Wang et al. [16] further extended the receptive field using dilated convolutions [17], enabling the model to capture larger context without increasing parameter count. Transformers [18] are increasingly applied to change detection due to their ability to model long-range dependencies. Unlike CNNs, which are limited by their local receptive fields, Transformers leverage self-attention mechanisms to capture global contextual information. Hybrid networks combining CNNs and Transformers, such as those by Chen et al. [19] and Li et al. [20], balance local and non-local attention and enhance change-related features through modules like discrepancy boosting and cross-temporal joint attention [21]. Pure Transformer architectures have also been explored, demonstrating competitive performance, while Zhang et al. [22] proposed computational optimizations to reduce the quadratic complexity of multi-head attention.

**Figure 1:**
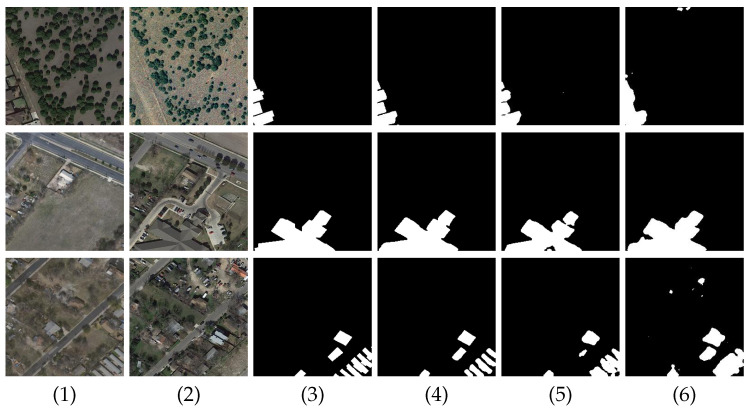
Three challenging RSCD scenarios from top to down have multiple adjacent objects, irregular objects, and fuzzy boundaries, respectively. The images from left to right are (**1**) input image T1, (**2**) input image T2, (**3**) ground truth (GT), (**4**) FARNet (ours), (**5**) SEIFNet [24], (**6**) STADE-CDNet [25]. These images are in the original RGB format of the LEVIR-CD dataset.

Although existing methods have shown success, they depend solely on RGB domain information. This limitation leads to reduced performance, as there is ambiguity between changed objects and the background in challenging scenarios, as illustrated in Figure 1. In frequency analysis, different frequency components indicate different characteristics of the object, which are more in a targeted manner. Different from the optical characteristics in the RGB domain, the frequency characteristics are helpful in locating the initial positions of different objects [23]. Inspired by this, we propose a frequency-aware refinement network (FARNet). This network initially identifies changed objects in the frequency domain and subsequently enhances their fine details within the RGB domain, effectively exploiting both frequency and RGB information to address ambiguity through a coarse-to-fine strategy.

First, a two-branch Transformer encoder is employed to extract multi-scale representations from bi-temporal images, enabling effective modeling of long-range dependencies and contextual information. Building upon these representations, we introduce a frequency-aware coarse localization stage, in which the frequency-aware module (FAM) is designed to leverage both high-frequency and low-frequency information to generate coarse localization maps. High-frequency components capture sharp changes, such as the edges and boundaries of changed objects, whereas low-frequency components represent large-scale structural changes. By integrating information from multiple frequency bands, the FAM provides a coarse but informative localization map that highlights potential change regions while preserving structural integrity. Following this, in the detail-preserving refinement fusion stage, the refinement fusion module (RFM) is designed to enhance the coarse localization results. RFM fuses the coarse localization maps with multi-scale contextual features derived from the Transformer encoder. This fusion enables the network to refine object details in the RGB domain by leveraging both spatial context and semantic cues. Through multi-scale attention and feature aggregation, RFM effectively sharpens the boundaries of changed regions and reduces false positives, ensuring that small or subtle changes are accurately delineated.

Finally, a lightweight segmentation head [26] is employed to decode the refined and fused features into the final change detection map. Additionally, to enhance the representation of high-frequency information, an edge loss [27] term is incorporated during training. This loss emphasizes the importance of edge regions, guiding the network to focus on boundaries and fine structural details, which further improves the precision of change detection. The main contributions of this work are summarized as follows:

1. We propose a novel framework termed FARNet, which effectively integrates frequency-domain analysis with RGB-domain refinement to improve change detection performance.
2. We design a frequency-aware module (FAM) to perform coarse localization of changed objects by exploiting frequency-domain representations, providing spatial priors for subsequent refinement.
3. We design a refinement fusion module (RFM) that integrates coarse localization maps with multi-scale contextual features, enhancing edge and structural information of changed objects through RGB-domain detail refinement.

## 2. Related Works

### 2.1. CNN-Based Methods

The inherent weight-sharing mechanism of CNN further establishes them as a cornerstone in processing remote sensing data. Building upon the architectural innovations in CNN-based change detection, fully convolutional networks (FCNs) have become prevalent for their end-to-end feature extraction capabilities in pixel-level analysis. Notably, Peng et al. [28] and Fang et al. [29] develop dense skip connection mechanisms that address the semantic gap through multi-scale feature fusion while substantially mitigating localization errors in deep feature maps. Furthermore, Ji et al. [30] implement a multi-scale perception strategy using dilated convolutions with varying receptive fields, achieving simultaneous detection of both micro-scale and macro-scale changing objects through hierarchical feature modeling.

Recent research has introduced attention mechanisms to address these limitations, dynamically refining feature focus to enhance adaptability to imaging condition changes and robustness to spatial transformations. Among these mechanisms, channel attention [31,32,33] adjusts channel weights of feature maps to emphasize specific spectral bands or features dynamically. Spatial attention [34,35] enhances focus on key changed objects by reducing attention to irrelevant background regions. Some studies [24,36,37,38,39] have combined channel and spatial attention to capture more distinctive changed objects.

### 2.2. Transformer-Based Methods

Transformer architecture has set a new standard in natural language processing with its impressive performance and capabilities. At its core, the Transformer captures positional dependencies in sequences using self-attention mechanisms and positional encoding. Vision Transformer (ViT) [40] expanded this concept into the field of computer vision.

Given the effectiveness of Transformers in the visual domain, many studies have applied them. SiamMixFormer [41] is a representative model in this field. SiamMixFormer adopts a novel design that transforms change features from the previous moment into query vectors and those from the subsequent moment into key-value pairs. Using self-attention, it effectively integrates bi-temporal features to enhance change detection accuracy. The Swin Transformer [42] has a powerful local window self-attention mechanism. SwinSUNet [43] and Feature Transformer Network (FTN) [44] both utilize the Swin Transformer as a core component to improve change information extraction, but they differ in their decoding strategies. SwinSUNet employs Swin Transformer units in the decoding phase to iteratively refine change features, thereby capturing changed object details more effectively. FTN, by contrast, employs a progressive attention feature pyramid design.

In recent years, to enhance the efficiency of tasks such as RSCD, researchers have developed a variety of hybrid models. In this context, several innovative models [25,45,46,47] have emerged, which are designed to integrate the strengths of CNN with Transformers. For example, Liu et al. [48] introduce an agricultural land change detection framework that combines CNN-based encoders and Transformer-based decoders. Feng et al. [49] present two attention-guided cross-scale fusion modules, emphasizing fine-grained features from CNNs and context-aware features from Transformers. These modules ensure effective information exchange between these two types of features at the same spatial resolution, thereby enhancing the understanding of complex change scenarios.

### 2.3. Frequency-Domain and Frequency–Spatial Hybrid Networks

Frequency-domain modeling has proven effective for remote sensing imagery, since high-frequency components encode fine boundaries while low-frequency components capture global semantics. AFENet [50] adaptively decouples RGB inputs into high- and low-frequency distributions for content-aware feature enhancement, and FSDENet [51] applies the Fast Fourier Transform (FFT) to perform global frequency mapping that jointly enhances frequency- and spatial-domain details. For change detection specifically, FSG-Net [52] introduces a frequency–spatial synergistic gated architecture that fuses the two domains via a learnable gate, achieving state-of-the-art results on high-resolution RSCD benchmarks. However, its gating operates at a single scale and relies on a relatively heavy decoder.

In contrast, our method adopts a coarse-to-refinement paradigm tailored for RSCD. Specifically, the proposed frequency-aware module (FAM) performs coarse localization of changed regions in the frequency domain by jointly exploiting high-frequency and low-frequency representations, while the refinement fusion module (RFM) further restores spatial details and structural consistency in the RGB domain. This design not only leverages the localization capability of frequency representations but also effectively compensates for the spatial information loss introduced during frequency transformation.

### 2.4. Contextual Refinement Techniques

Context modeling is critical for suppressing pseudo-changes caused by illumination, seasonal variation, and registration noise. Transformer-based methods such as BIT [19], ChangeFormer [53], and SwinSUNet [43] capture long-range dependencies through self-attention, while DSIFN [36] and SNUNet [29] strengthen cross-temporal context via deep difference fusion and dense skip connections. These designs, however, are either computationally heavy or operate purely in the spatial domain, which limits their ability to recover boundaries lost in the frequency domain. Our refinement fusion module (RFM) instead operates on frequency–spatial decoupled features and selectively re-injects high-frequency cues into the low-frequency semantic context, refining both the what and where of detected changes at a substantially lower cost.

## 3. Methodology

### 3.1. Overall Structure

**Figure 2:**
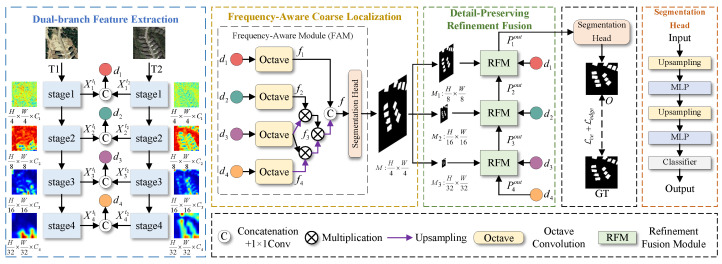
Overall structure of the FARNet. First, in the feature extraction stage, bi-temporal multi-level features are extracted from the image pair using a weight-sharing Transformer encoder. Then, in the frequency-aware coarse localization stage, the bi-temporal multi-level features are fed into the FAM to generate a coarse localization map *M*. Next, in the detail-preserving refinement and fusion stage, the bi-temporal multi-level features together with the coarse localization map *M* are input into the RFM to obtain the refined feature map $P_{1}^{out}$. Finally, $P_{1}^{out}$ is fed into the segmentation head to produce the change map, where white indicates changed areas and black indicates unchanged areas.

In this paper, we propose a FARNet for RSCD. As shown in Figure 2, in the dual-branch feature extraction stage, the bi-temporal input images $\{T_{1},T_{2}\in R^{H\times W\times 3\}}$ are processed through a weight-shared Siamese Transformer encoder, generating multi-scale hierarchical features represented as ${\left\{X_{i}^{t_{1}},X_{i}^{t_{2}}\in R^{\frac{H}{2^{i+1}}\times \frac{W}{2^{i+1}}\times C_{i}}\right\}}_{i=1}^{4}$, where $C_{i}$ takes values of 32, 64, 160, and 256 for i=1,…,4, respectively. Subsequently, we used concatenation followed by convolutional operations to generate the temporal difference features with a unified channel dimension of C=64 from the bi-temporal features ${\left\{d_{i}\in R^{\frac{H}{2^{i+1}}\times \frac{W}{2^{i+1}}\times C}\right\}}_{i=1}^{4}$. Afterwards, in the frequency-aware coarse localization stage, given $d_{i}$ as input, we design the frequency-aware module (FAM), which first extracts frequency domain features from $d_{i}$ and then obtains the coarse localization map $M\in R^{H\times W}$ through the segmentation head. Then, in the detail-preserving refinement fusion stage, *M* and $d_{i}$ are given as inputs. We design the refinement fusion module (RFM), under the guidance of *M*, to progressively achieve feature $d_{i}$ correction and cross-layer fusion. Finally, the fusion feature extracted from the RFM is input into the decoder to generate the anticipated change map $O\in R^{H\times W}$.

The FAM effectively decouples high-frequency and low-frequency information, thereby enhancing feature discrimination. The RFM captures global and local dependencies, improving semantic consistency. The lightweight segmentation head [26] ensures efficient reconstruction while preserving fine details, and the edge-aware loss [27] algorithm explicitly guides boundary refinement. Together, these components work synergistically, enabling FARNet to achieve superior performance in high-resolution remote sensing change detection by enhancing detail preservation, boundary accuracy, and overall feature representation.

### 3.2. Frequency-Aware Module

As described in [54], octave convolution (Octave) can learn to decompose images into low-frequency and high-frequency components in the frequency domain. Low-frequency features correspond to pixels with gentle intensity variations, such as large homogeneous color regions, and they typically represent the main body of objects. In contrast, high-frequency components refer to pixels with strong intensity changes, such as object edges in images. Inspired by this, and to effectively resolve the semantic ambiguity between changed objects and the background in the RGB domain, we design FAM. FAM is a frequency-guided coarse localization module. This module automatically decomposes features into high-frequency and low-frequency parts to form a frequency-domain representation of changed objects, and then, it progressively fuses multi-scale frequency representations from bi-temporal images to generate an initial change localization map. The detailed process is shown in Figure 2.

**Figure 3:**
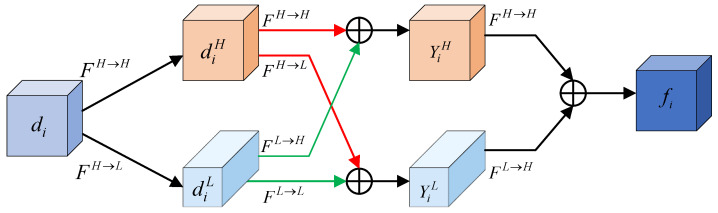
Architecture of octave convolution (Octave). Green arrows correspond to information updating, while red arrows facilitate information exchange between the two frequencies.

Specifically, we first employ octave convolution [54] to enable the network to automatically focus on both high-frequency and low-frequency information, which enables FARNet to simultaneously capture detailed and structural features. As shown in Figure 3, the detailed process of the octave convolution output $Y_{i}=\left\{Y_{i}^{H},Y_{i}^{L}\right\}$ is described as follows:

(1) $$Y_{i}^{H}=F\left(d_{i}^{H};W^{H\to H}\right)+\mathrm{Upsample}\left(F\left(d_{i}^{L};W^{L\to H}\right)\right),$$

(2) $$Y_{i}^{L}=F\left(d_{i}^{L};W^{L\to L}\right)+F\left(\mathrm{pool}\left(d_{i}^{H}\right);W^{H\to L}\right),$$

where F(X;W) represents a convolutional operation with learnable parameters *W*, Upsample(·) denotes an upsampling operation, and pool(·) denotes an average pooling operation.

Considering that high-frequency textures and low-frequency contour attributes are both crucial for localizing changed objects, so we integrate them into a unified frequency domain representation:

(3) $$f_{i}=Y_{i}^{H}⊕\mathrm{Upsample}\left(Y_{i}^{L}\right),$$

where ⊕ denotes elementwise addition.

Then, as shown in Figure 2, deep frequency domain features are progressively integrated to better utilize the semantic relationships between adjacent layers. In addition to modeling high-level feature associations, we leverage high-resolution information from the first layer to enhance details. The process is as follows:

(4) $$f_{d}=\mathrm{Upsample}\left(f_{3}⊗\mathrm{Upsample}\left(f_{4}\right)\right)⊗(f_{2}⊗\mathrm{Upsample}\left(f_{3}\right)),$$

(5) $$f=\mathrm{cat}(\mathrm{Upsample}\left(f_{d}\right),f_{1}),$$

where ⊕ denotes elementwise addition, ⊗ denotes elementwise multiplication, and cat(·) represents concatenation along with a 1×1 convolution.

Finally, the feature map *f* is upsampled to increase its size and serve as the input to the multilayer perceptron (MLP). By repeating the operations of upsampling and MLP, detailed information of the image is gradually restored and expressed as follows:

(6) $$M={\mathrm{Conv}}_{3\times 3}\left(\mathrm{MLP}\left(\mathrm{Upsample}\left(\mathrm{MLP}\left(\mathrm{Upsample}\left(f\right)\right)\right)\right)\right),$$

where ${\mathrm{Conv}}_{3\times 3}$ denotes the convolution operation with a kernel size of 3×3. We use simple convolution to obtain a localization change map *M*, which reveals the initial location of the object that has changed.

### 3.3. Refinement Fusion Module

**Figure 4:**
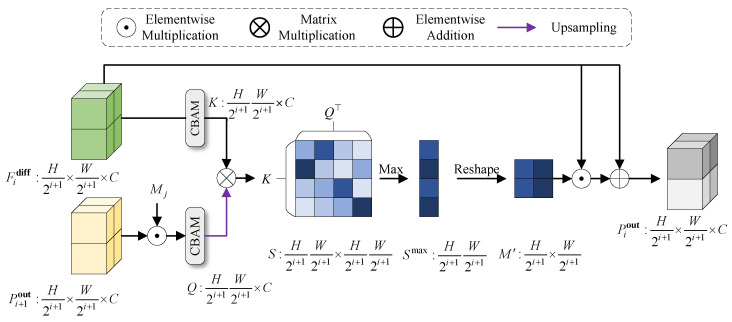
Architecture of refinement fusion module (RFM).

In the previous section, we introduced the use of frequency domain features for the coarse localization of changed objects. Although this stage can roughly detect the changed objects, the precision and completeness of localization are still insufficient. Considering that the high-resolution details in the RGB domain provide more spatial information than frequency-domain features, they enable the network to better adjust the segmentation boundaries to conform to the actual object contours and edges. Therefore, we design an RFM that leverages detailed RGB domain information to correct and refine segmentation boundaries. The process is shown in Figure 4.

We first reduce the number of input features ${\left\{X_{i}^{t_{1}},X_{i}^{t_{2}}\right\}}_{i=1}^{4}$ channels to 64, denoted as ${\left\{F_{i}^{t_{1}},F_{i}^{t_{2}}\right\}}_{i=1}^{4}$, which improves computational efficiency while still retaining relevant information for detection. Then, we extract multi-level feature differences ${\left\{F_{i}^{\mathrm{diff}}\right\}}_{i=1}^{4}$ from the bi-temporal features ${\left\{F_{i}^{t_{1}},F_{i}^{t_{2}}\right\}}_{i=1}^{4}$. This can be specifically expressed as follows:

(7) $$F_{i}^{\mathrm{diff}}=\mathrm{Relu}(\mathrm{BN}\left({\mathrm{Conv}}_{3\times 3}\left(\mathrm{cat}\left(F_{i}^{t_{1}},F_{i}^{t_{2}}\right)\right)\right)),$$

where cat(·) represents concatenation along the channel dimension, BN represents the normalization operation, and ReLU represents the activation function.

In order to make full use of the existing coarse localization map *M*, the coarse localization map is resized to produce three coarse localization maps ${\left\{M_{j}\in R^{\frac{H}{2^{j+2}}\times \frac{W}{2^{j+2}}}\right\}}_{j=1}^{3}$ of different scales. Given that ${\left\{P_{i}^{\mathrm{out}}\in R^{\frac{H}{2^{i+1}}\times \frac{W}{2^{i+1}}}\right\}}_{i=1}^{4}$ denotes the output of RFM, where $P_{4}^{\mathrm{out}}$ represents $F_{4}^{\mathrm{diff}}$. We first multiply the feature maps ${\left\{P_{i}^{\mathrm{out}}\right\}}_{i=2}^{4}$ by the coarse maps $M_{j}$, a corrective guide that is particularly beneficial when objects blend with their surroundings. Then, we use CBAM [55] to adaptively focus on important features. Subsequently, by upsampling and reshaping, the feature map $Q\in R^{\frac{H}{2^{i+1}}\frac{W}{2^{i+1}}\times C}$ is obtained. Meanwhile, the feature ${\left\{F_{i}^{\mathrm{diff}}\right\}}_{i=1}^{3}$, after being processed by CBAM, is reshaped into $K\in R^{\frac{H}{2^{i+1}}\frac{W}{2^{i+1}}\times C}$. This process is calculated by the following:

(8) $$K=\mathrm{Reshape}(\mathrm{CBAM}\left(F_{i}^{\mathrm{diff}}\right)),$$

(9) $$Q=\mathrm{Reshape}(\mathrm{Upsample}\left(\mathrm{CBAM}\left(P_{i+1}^{\mathrm{out}}⊙M_{j}\right)\right)),$$

where ⊙ denotes elementwise multiplication, and Upsample(·) represents an upsampling operation.

To fully integrate multi-level features and feed deep semantic information back into shallow features, we capture the correlation between cross-level features by calculating the similarity of the same pixel across feature maps at different scales. This approach effectively promotes interactions and the sharing of information. Finally, the feature similarity map *S* is calculated as follows:

(10) $$S=KQ^{⊤},$$

where $S\in R^{\frac{H}{2^{i+1}}\frac{W}{2^{i+1}}\times \frac{H}{2^{i+1}}\frac{W}{2^{i+1}}}$, and *⊤* denotes matrix transpose operator.

We select the maximum similarity value for each row of pixels in *S*. This process is calculated by the following:

(11) $$S^{\max}=\max_{i=1\ldots HW}S\left[:,i\right],$$

where $S^{\max}\in R^{\frac{H}{2^{i+1}}\frac{W}{2^{i+1}}}$.

Afterwards, we reshape $S^{\max}$ into $M^{\prime }\in R^{\frac{H}{2^{i+1}}\times \frac{W}{2^{i+1}}}$ to enhance the attention of the network on changing objects. Finally, the output of the proposed RFM is as follows:

(12) $$P_{i}^{\mathrm{out}}=M^{\prime }⊙F_{i}^{\mathrm{diff}}⊕F_{i}^{\mathrm{diff}},$$

where ⊙ denotes elementwise multiplication, and ⊕ denotes elementwise addition.

### 3.4. Loss Functions

We use cross-entropy loss [53] and edge loss [27] to optimize the network’s parameters. Cross-entropy loss effectively guides the network to learn global discriminative features that help distinguish between different classes. Edge loss focuses on optimizing the performance of the network on object boundaries by penalizing errors in the edge regions. In cases where class boundaries are blurred, edge loss is particularly effective, significantly enhancing high-frequency details. The calculation formulas for the loss functions are as follows:

(13) $$L=\alpha L_{ce}+\left(1-\alpha \right)L_{edge},$$

where L denotes the loss between the final change map *O* and ground truth, $L_{ce}$ represents the cross-entropy loss function, $L_{edge}$ represents edge loss, and α is a hyperparameter used to balance cross-entropy loss and edge loss.

## 4. Experiments

### 4.1. Datasets

FARNet uses supervised learning and requires labeled change detection datasets for training. The experiments were performed on three benchmark datasets. The WHU-CD dataset [30] comprises a pair of aerial images at 0.2 m resolution with dimensions of 32,507 × 15,354 pixels. The dataset covers a city in New Zealand and focuses on detecting changes in buildings. These images were cropped into 256 × 256 patches, generating 5947 training, 743 validation, and 744 testing samples. The LEVIR-CD dataset [56] contains 637 pairs of high-resolution Google Earth images, each with 0.5 m resolution and 1024 × 1024 pixel dimensions. After patch cropping (256 × 256), we obtained 7120 training, 1024 validation, and 2048 testing samples. The dataset covers multiple cities in TX, USA, including 20 different areas. The LEVIR-CD+ dataset [56] is a large-scale building change detection benchmark built upon LEVIR-CD. It includes 985 image pairs with roughly 80,000 building change instances, posing a substantial challenge for large-scale change detection. Non-overlapping 256 × 256 patches were cropped, yielding 10,192 training and 5568 testing samples.

### 4.2. Implementation Details

In the experiments, the network was trained using Python 3.9.20 and the PyTorch 1.7.1 framework on two NVIDIA RTX 2080Ti GPUs. The AdamW optimizer was used with the following settings: a batch size of 16, an initial learning rate of 0.0001, momentum of 0.99, epochs of 200, and weight decay of 0.01. A linear learning rate decay strategy was implemented. To ensure the reproducibility of the experiments, we fixed the random seed to 42 in all experiments and will set this in the code. Data augmentation was applied to all experimental data during the training phase to improve robustness and generalization. The techniques included flipping, cropping, and Gaussian blurring.

### 4.3. Evaluation Metrics

In the evaluation phase, five widely used metrics are employed: precision (Pre), recall (Rec), F1 score (F1), intersection over union (IoU), and overall accuracy (OA). Their definitions are as follows:

(14) Pre=TP/(TP+FP),

(15) Rec=TP/(TP+FN),

(16) F1=2×Rec×Pre/(Rec+Pre),

(17) IoU=TP/(TP+FP+FN),

(18) OA=(TP+TN)/(TP+TN+FP+FN),

where TP, TN, FP, and FN denote the number of true positives, true negatives, false positives, and false negatives, respectively. The five evaluation metrics take values ranging from 0 to 1, with higher values indicating better network performance. Notably, the IoU and F1 better reflect the detection capability of the network in the change detection task.

### 4.4. Compared Methods

To validate the effectiveness of FARNet for RSCD, we selected nine state-of-the-art networks, which are as follows:

1. FC-EF [13] concatenates two input images from different timestamps into a multi-channel image, which is processed by the FCN for feature extraction and change detection.
2. FC-Siam-Diff [13] is built upon FC-EF and extracts multi-level features from bi-temporal images. It applies difference operations to generate a difference map, emphasizing regions of change. Finally, it uses a twin network with shared weights to fuse these features for more accurate change detection.
3. FC-Siam-Conc [13] is based on FC-EF and uses a twin network with shared weights for extracting multi-level features. This network combines bi-temporal features through concatenation.
4. BIT [19] uses ResNet-18 [57] to extract high-level features from bi-temporal images, mapping them into a semantic space. It then leverages the encoder–decoder structure of Transformers for global context modeling. Finally, the enriched semantic representations are mapped back to the pixel space, and difference operations are applied to highlight change regions.
5. ChangFormer [53] is a Transformer-based twin network architecture. Through multi-scale feature modeling, it captures remote sensing details more effectively, improving change detection performance.
6. DMINet [21] extracts multi-level features using ResNet-18 and concatenates them channel-wise. It uses a joint attention module to learn spatiotemporal relationships across different feature levels. Finally, it provides multi-level supervision through a feature decoder to enhance the detection of changed objects.
7. SEIFNet [24] employs a twin hierarchical backbone network to capture multi-level image information through feature maps. It uses attention mechanisms to extract global and local information from bi-temporal features, highlighting changed objects. Finally, a fusion module integrates cross-level features to enhance change detection accuracy.
8. STADE-CDNet [25] uses a Transformer encoder to extract low-to-high-level semantic information from bi-temporal images, capturing temporal dependencies. A memory module stores temporal information, and a difference enhancement module emphasizes change information, improving change detection performance.
9. SDA-Encoding [58] employs a wavelet transform to decompose features into low-frequency and high-frequency components. In the high-frequency branch, local details and boundary information are enhanced through spatial pyramid pooling and cross-scale alignment fusion. In the low-frequency branch, coordinate attention and selective fusion attention are used to restore positional information and model region-level contextual dependencies. Finally, the two frequency branches are complementarily fused in the spatial domain to achieve unified modeling of both fine-grained and structural changes.

### 4.5. Results and Discussion

#### 4.5.1. Results on WHU-CD Dataset

Table 1 provides the quantitative results of various methods on the WHU-CD dataset. FARNet achieves the best results across five commonly used evaluation metrics. Notably, its IoU and F1 are recorded at 90.00% and 94.74%, respectively, outperforming all other methods.

**Figure 5:**
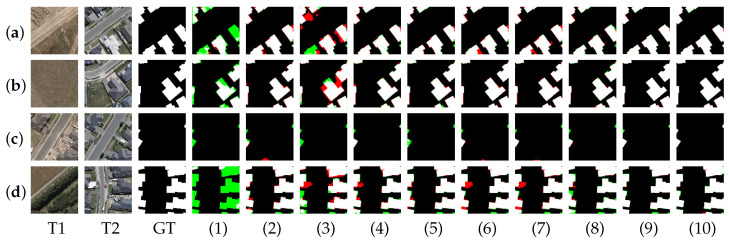
The visualization results achieved by different change detection methods on the WHU-CD test set. Subfigures (**a**–**d**) illustrate the prediction results of all competing methods corresponding to different sample images. Columns (1)–(10) show the predicted change maps of FC-EF, FC-Siam-Diff, FC-Siam-Conc, BIT, ChangeFormer, DMINet, SEIFNet, STADE-CDNet, SDA-Encoding, and FARNet. For the color scheme, white represents true positive predictions, black represents true negatives, red refers to false positives, and green indicates false negatives.

Figure 5 presents the visualization comparison results on the WHU-CD dataset. In Figure 5a,b, FARNet demonstrates its ability to accurately segment the changed objects, highlighting its effectiveness in change detection. In contrast, other methods generate a higher number of false detections and missed detections, particularly in the edge regions where segmentation is more challenging. As shown in Figure 5c, when multiple changed objects are very small, FARNet is the only method capable of detecting all changes accurately, demonstrating its superiority in detecting fine-grained changes. However, despite these strengths, FARNet still faces certain limitations. For instance, as shown in Figure 5d, in extremely complex scenarios with highly cluttered backgrounds and subtle changes, some false detections may still occur. While it is improved compared to other methods, it may not be perfect in all cases.

#### 4.5.2. Results on LEVIR-CD Dataset

Table 2 provides the quantitative results of various methods on the LEVIR-CD dataset. FARNet outperforms other methods across four key evaluation metrics. Although its precision is 1.02% lower than the top-ranked SDA-Encoding, it surpasses SDA-Encoding in recall by 1.25%. Additionally, FARNet achieves IoU and F1 values that are 0.23% and 0.13% higher, respectively, than those of the second-ranked SEIFNet.

**Figure 6:**
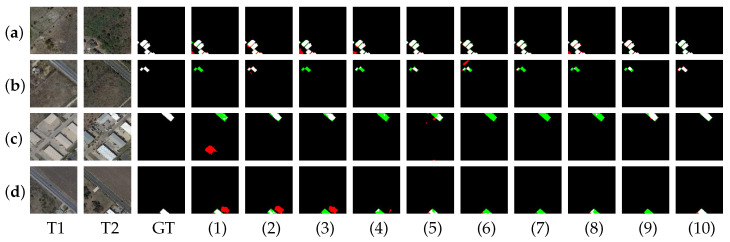
The visualization results achieved by different change detection methods on the LEVIR-CD test set. Subfigures (**a**–**d**) illustrate the prediction results of all competing methods corresponding to different sample images. Columns (1)–(10) show the predicted change maps of FC-EF, FC-Siam-Diff, FC-Siam-Conc, BIT, ChangeFormer, DMINet, SEIFNet, STADE-CDNet, SDA-Encoding, and FARNet. For the color scheme, white represents true positive predictions, black represents true negatives, red refers to false positives, and green indicates false negatives.

Figure 6 presents the visualization comparison results on the LEVIR-CD dataset. In Figure 6a, in complex scenes where changed objects are challenging to distinguish from the background in the RGB domain, FARNet is the only method that accurately segments all changed objects. In Figure 6b, due to ambiguity between the changed objects and the background, only FC-Siam-Di and FARNet successfully detect changes in small buildings, whereas other methods fail to capture these subtle changes effectively. In Figure 6c,d, most methods fail to detect the changed buildings effectively, further highlighting that FARNet can effectively distinguish changed objects from the background.

#### 4.5.3. Results on LEVIR-CD+ Dataset

Table 3 provides the quantitative results of various methods on the LEVIR-CD+ dataset. FARNet outperforms other methods across three key metrics, demonstrating its superior performance in addressing the challenges of dataset. On the more challenging LEVIR-CD+ dataset, FARNet achieved the highest scores in the most critical indicators, namely IoU and F1. It surpassed BIT by 2.66% and 1.75%, respectively. FARNet also demonstrated satisfactory performance in terms of precision and recall.

**Figure 7:**
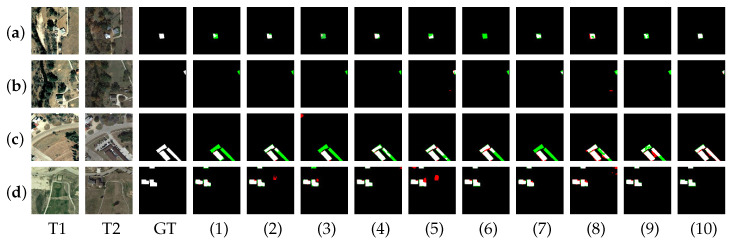
The visualization results achieved by different change detection methods on the LEVIR-CD+ test set. Subfigures (**a**–**d**) illustrate the prediction results of all competing methods corresponding to different sample images. Columns (1)–(10) show the predicted change maps of FC-EF, FC-Siam-Diff, FC-Siam-Conc, BIT, ChangeFormer, DMINet, SEIFNet, STADE-CDNet, SDA-Encoding, and FARNet. For the color scheme, white represents true positive predictions, black represents true negatives, red refers to false positives, and green indicates false negatives.

Figure 7 presents the visualization comparison results on the LEVIR-CD+ dataset. In Figure 7a–c, in bi-temporal images with background interference, only FARNet can detect and clearly segment all the changed objects. In Figure 7d, FARNet produces fewer green and red pixels, clearly segmenting the changed objects. These results indicate that FARNet significantly reduces false positives and false negatives caused by irrelevant noise. Finally, the generalization capability across different datasets or unseen regions requires further validation, which could be addressed in future work.

#### 4.5.4. Ablation Studies

To determine the contribution of each component within FARNet, ablation studies were performed on both the LEVIR-CD and WHU-CD datasets. The experimental outcomes, detailed in Table 4 and Table 5, were obtained by systematically removing individual components and measuring the resulting performance fluctuations. These results offer insights into the relative importance of each component to the overall effectiveness of FARNet.

**Figure 8:**
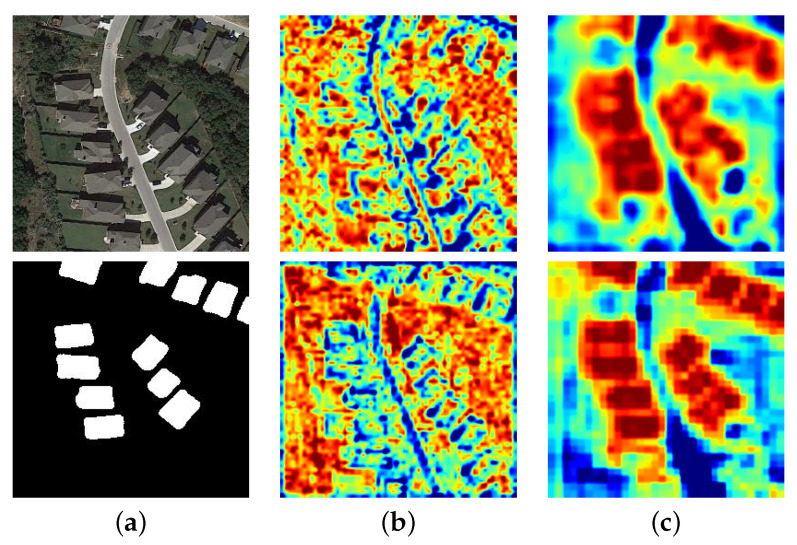
An example visualization of the main modules in the network. (**a**) The original input T2 and the predicted CD map; (**b**) the input and output of FAM; (**c**) the input and output of RFM.

**Figure 9:**
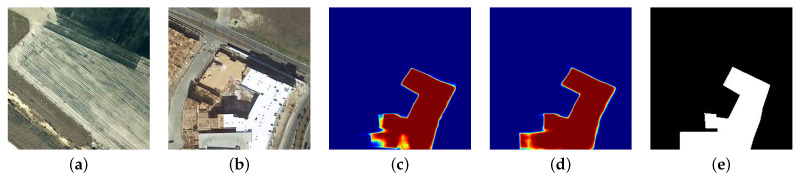
An example of visualizing the edge loss. (**a**) Input image T1. (**b**) Input image T2. (**c**) *w*/*o* edge loss. (**d**) With edge loss. (**e**) Ground truth.

1. Ablation Study of Frequency-Aware Module (FAM): To address the detection ambiguity caused by the similarity of features between changed objects and the background in the RGB domain in remote sensing scenarios, the FAM module is designed as follows: (1) Employ octave convolution to explicitly separate high-frequency details from low-frequency contour features, thereby enhancing boundary discrimination for similar targets; (2) generate a prior for the changed objects through coarse localization, guiding the subsequent refinement module to focus on key areas. As shown in Table 4, on the LEVIR-CD dataset, FAM improved IoU by 0.69% and F1 by 0.40%; on the WHU-CD dataset, IoU increased by 0.51% and F1 by 0.28%, verifying its effectiveness. As shown in Figure 8b, after the application of octave convolution, these features more effectively represent the positional information of the changed areas.
2. Ablation Study of Refinement Fusion Module (RFM): To make the segmentation boundaries clearer, the RFM is designed as follows: (1) Introduce high-resolution features from the RGB domain and fuse differential features from adjacent layers using cross-layer channel attention mechanisms to enhance edge detail modeling; (2) use the coarse localization map for spatial constraints to generate refined segmentation results. As shown in Table 4, on the LEVIR-CD dataset, RFM improved IoU by 0.47% and F1 by 0.27%, and on the WHU-CD dataset, IoU increased by 0.46% and F1 increased by 0.26%, verifying its effectiveness. As shown in Figure 8c, the coarse localization map has located the changed areas, but the segmentation boundaries are still blurred. After RFM processing, the feature map mainly focuses on the changed areas, significantly reducing noise interference on the segmentation boundaries.
3. Ablation Study of Loss Function Weights: We aim to assess the impact of the loss function coefficients on the performance of FARNet using the LEVIR-CD dataset. The goal of these experiments was to understand how adjusting the coefficients affects overall performance. The results in Table 5 show that the best performance was achieved with cross-entropy and edge loss coefficients set to 0.9 and 0.1, respectively. This suggests that edge loss enhances high-frequency details, improving the ability of the network to detect changes more accurately. Thus, subsequent experiments used a cross-entropy coefficient of 0.9. Although results for α in the range of 0.1 to 0.4 were not included, preliminary experiments indicate that performance in this lower range is significantly worse, and therefore has limited practical relevance. As shown in Figure 9c, without edge loss, the boundaries of the changed areas are blurrier. In contrast, as shown in Figure 9d, with the addition of edge loss, the boundaries become clearer, enabling the model to more accurately distinguish the changed objects.

To further evaluate the robustness of FARNet under challenging real-world conditions, additional experiments were conducted on the LEVIR-CD dataset by introducing noise corruption and cloud occlusion into the testing images. As shown in Table 6, FARNet maintains competitive performance under both challenging conditions. Under noise corruption, the proposed method achieves 84.48% IoU and a 91.58% F1 score, while under cloud occlusion, it still obtains an IoU of 83.93% and F1 score of 91.27%. Although slight performance degradation can be observed compared with the original testing setting, the decrease remains relatively limited, demonstrating the robustness and generalization capability of the proposed framework in complex real-world scenarios.

#### 4.5.5. Comparison of Complexity

As shown in Table 7, FARNet has 55.92 M parameters and requires 41.84 G FLOPs, which are higher than other methods. Nevertheless, this additional complexity leads to superior change detection performance, demonstrating the effectiveness of the proposed frequency–RGB dual-domain fusion mechanism.

#### 4.5.6. Discussion

For the RSCD task, this paper introduces a coarse-to-fine frequency refinement network (FARNet), which consists primarily of two stages. To effectively address the ambiguity between changed objects and the background in the RGB domain, we design the frequency-aware module (FAM) in the frequency-aware coarse localization stage, which leverages frequency domain information to help the network locate the initial positions of changed objects. To enable precise segmentation of changed objects, we design the refinement fusion module (RFM) in the detail-preserving refinement fusion stage, which refines and corrects the information of changed objects using RGB domain information. Additionally, edge loss is introduced to improve the ability of network to capture and optimize high-frequency details, specifically targeting the edge regions of the image. FARNet outperforms other advanced algorithms on the WHU-CD, LEVIR-CD, and LEVIR-CD+ datasets by excelling in changed object recognition and showing resilience to interference. However, this study solely focused on identifying building changes. In the future, we plan to explore the identification of multi-category changed objects in greater depth. Also, we plan to explore weakly supervised learning strategies to reduce reliance on fine-grained annotations and further investigate the potential of frequency-domain features in RSCD. Specifically, in weakly supervised settings, model training often relies on pseudo-labels, and frequency-domain features have a natural advantage in distinguishing real structural changes from low-frequency noise. Therefore, pseudo-labels generated based on frequency-domain information are generally cleaner and more reliable than those relying solely on spatial-domain features, which is expected to further improve the performance ceiling of weakly supervised learning.

## 5. Conclusions

In this paper, we propose a frequency-aware refinement network (FARNet) to resolve the ambiguity between changed objects and the background in the RGB domain by incorporating frequency domain information. Specifically, a frequency-aware module (FAM) is designed to automatically aware frequency information, guiding the network to produce a coarse localization map during the frequency-aware coarse localization stage. Then, a refinement fusion module (RFM) is designed to utilize the coarse localization map and RGB domain information to achieve progressive correction and fusion of multi-scale features during the detail-preserving refinement fusion stage. Finally, an edge loss function optimizes segmentation results by focusing on high-frequency features. The experimental results demonstrate that FARNet outperforms state-of-the-art algorithms on the WHU-CD, LEVIR-CD, and LEVIR-CD+ datasets. The IoU for each dataset is 90.00%, 85.52%, and 75.65%, and the F1 for each dataset is 94.74%, 92.19%, and 86.13%, respectively.

## Figures and Tables

**Table 1:** Generalization experiments conducted on the WHU-CD dataset (with the best results indicated in bold).

Methods	OA (%)	IoU (%)	F1 (%)	Pre (%)	Rec (%)
FC-EF	98.19	58.30	73.66	87.25	63.73
FC-Siam-Diff	98.25	67.34	80.48	72.27	90.80
FC-Siam-Conc	95.91	45.37	62.42	49.08	85.72
BIT	98.75	72.39	83.98	86.64	81.48
ChangeFormer	99.11	79.16	88.37	92.21	84.83
DMINet	98.97	79.68	88.69	93.84	86.25
SEIFNet	98.90	76.04	86.39	87.01	85.77
STADE-CDNet	99.20	80.77	89.36	94.06	85.11
SDA-Encoding	99.59	89.87	94.67	96.69	**92.73**
FARNet(ours)	**99.59**	**90.00**	**94.74**	**97.50**	92.13

**Table 2:** Generalization experiments conducted on the LEVIR-CD dataset (with the best results indicated in bold).

Methods	OA (%)	IoU (%)	F1 (%)	Pre (%)	Rec (%)
FC-EF	98.38	72.19	83.85	85.10	82.63
FC-Siam-Diff	98.98	81.38	89.74	92.09	87.50
FC-Siam-Conc	98.62	75.72	86.18	87.83	84.59
BIT	98.92	80.68	89.31	89.24	89.37
ChangeFormer	99.04	82.48	90.40	92.05	88.80
DMINet	99.07	82.99	90.71	92.52	89.95
SEIFNet	99.09	83.40	90.95	92.49	89.46
STADE-CDNet	98.91	80.44	89.16	90.65	87.72
SDA-Encoding	99.20	85.29	92.06	**93.34**	90.82
FARNet (ours)	**99.21**	**85.52**	**92.19**	92.32	**92.07**

**Table 3:** Generalization experiments conducted on the LEVIR-CD+ dataset (with the best results indicated in bold).

Methods	OA (%)	IoU (%)	F1 (%)	Pre (%)	Rec (%)
FC-EF	97.63	56.05	71.83	69.75	74.05
FC-Siam-Diff	98.61	70.83	82.93	83.18	82.68
FC-Siam-Conc	98.17	63.89	77.97	76.38	79.63
BIT	98.77	72.99	84.38	**87.57**	81.42
ChangeFormer	98.61	70.74	82.86	83.30	82.44
DMINet	98.68	71.19	83.17	86.21	80.33
SEIFNet	98.54	69.22	81.81	82.86	80.79
STADE-CDNet	98.53	71.02	83.06	78.07	**88.73**
SDA-Encoding	98.84	74.97	85.70	85.81	85.58
FARNet(ours)	**98.87**	**75.65**	**86.13**	86.12	86.15

**Table 4:** Ablation study of different modules (with the best results indicated in bold). ✓ indicates presence, × indicates absence.

FAM	RFM	LEVIR-CD			WHU-CD		
		**OA (%)**	**IoU (%)**	**F1 (%)**	**OA (%)**	**IoU (%)**	**F1 (%)**
×	✓	99.18	84.83	91.79	99.57	89.49	94.46
✓	×	99.18	85.05	91.92	99.57	89.54	94.48
✓	✓	**99.21**	**85.52**	**92.19**	**99.59**	**90.00**	**94.74**

**Table 5:** Multiple α values were used for performance comparison on the LEVIR-CD dataset (with the best results indicated in bold).

α	LEVIR-CD				
	**OA (%)**	**IoU (%)**	**F1 (%)**	**Pre (%)**	**Rec (%)**
1.0	99.18	84.98	91.88	92.77	91.01
0.9	**99.21**	**85.52**	**92.19**	92.32	**92.07**
0.8	99.18	85.01	91.90	**92.95**	90.86
0.7	99.18	84.98	91.88	92.56	91.21
0.6	99.15	84.57	91.64	91.89	91.39
0.5	99.17	84.84	91.80	92.66	90.95

**Table 6:** The experimental results of different challenging conditions on the LEVIR-CD dataset (with the best results indicated in bold).

Operation	LEVIR-CD				
	**OA (%)**	**IoU (%)**	**F1 (%)**	**Pre (%)**	**Rec (%)**
Noise corruption	99.15	84.48	91.58	**92.63**	90.56
Cloud occlusion	99.12	83.93	91.27	91.82	90.72
-	**99.21**	**85.52**	**92.19**	92.32	**92.07**

**Table 7:** Complexity comparison of different methods.

Methods	Para. (M)	FLOPs (G)
FC-EF	1.35	3.59
FC-Siam-diff	1.35	4.74
FC-Siam-conc	1.55	5.34
BIT	12.40	10.87
DMINet	6.24	14.55
STADE-CDNet	11.9	14.3
SEIFNet	27.91	8.37
SChanger	2.37	17.91
CASP	14.55	9.19
SDA-Encoding	45.57	44.80
FARNet (Ours)	55.92	41.84

## Data Availability

Three publicly available datasets were used in this study. The WHU-CD dataset can be found at https://study.rsgis.whu.edu.cn/pages/download/building_dataset.html (accessed on 1 January 2026). The LEVIR-CD and LEVIR-CD+ datasets can be found at https://justchenhao.github.io/LEVIR/ (accessed on 1 January 2026). The data presented in this study are available upon request from the corresponding author.
